# Comparative Analysis of Intellectual Quotient in Developmental Population with Severe Hearing Loss: Hearing Aids vs. Cochlear Implant Users

**DOI:** 10.3390/life14010012

**Published:** 2023-12-20

**Authors:** Arianna Di Stadio, Pietro De Luca, Valentina Ippolito, Paola Vedova, Sabina Garofalo, Rosaria Turchetta, Salvatore Ferlito, Antonio della Volpe

**Affiliations:** 1 GF Ingrassia Department, University of Catania, 95121 Catania, Italy; ferlito@unict.it; 2 Otolaryngology Department, Isola Tiberina—Gemelli Isola Hospital, 00186 Rome, Italy; dr.dlp@hotmail.it (P.D.L.); antoniodellavolpe@yahoo.it (A.d.V.); 3 ENT Department, Pediatric CI Regional Referral Centre, Santobono-Pausilipon Children’s Hospital of Naples, 80129 Naples, Italy; logopedistaippolitovalentina@gmail.com (V.I.); garofalo.sabina@libero.it (S.G.); 4 Neuropsychiatric Department, Santobono-Pausillipon Hospital, 80129 Naples, Italy; paolavedova1@gmail.com; 5 Pediatric Audiology Unit, Organ of Sense Department, University La Sapienza of Rome, 00185 Rome, Italy; rosaria.turchetta@uniroma1.it

**Keywords:** hearing loss, deafness, cochlear implant, hearing aid, children, cognition, intelligence, IQ

## Abstract

The development of language, memory and intellectual functions is linked to normal hearing and correct sounds interpretation. Hearing loss (HL), especially in its severe form, negatively affects the development of these functions. This prospective study aimed at comparing the Intelligent Quotients (IQ) of children with cochlear implants (CI) with the ones of people wearing hearing aids (HA) after one year of hearing rehabilitation. 21 subjects with severe/profound bilateral hearing loss (deafness) were included in this study. Eleven children with congenital profound HL underwent CI and ten children with moderate to severe HL (congenital and acquired) were rehabilitated by HA. Children’s IQs were assessed at enrolment (T0) and 12 months after hearing aids/CI use plus speech therapy. Statistical analyses were performed to analyze the data within and between groups. Comparison of IQs showed no statistically significant differences between CI and HA none at T0 and T1. The subtests showed lower scores in verbal comprehension and process speed index in patients treated with HA when compared to CI. This study showed that auditory rehabilitation can support the normal development of cognitive function in children between six and eight years of age. The use of the correct hearing aids based on the patient’s hearing thresholds is important to maximize the rehabilitation outcomes. Due to the small sample size, although stratified for age, our results must be considered preliminary and further analyses on larger samples are needed to confirm our data.

## 1. Introduction

Cognitive skills such as language, memory, reasoning, perceptual functioning, and visuomotor functions are essential for proper integration into society, and the loss of a sense such hearing can be a social barrier. The early rehabilitation of hearing loss is essential to avoid difficulties in cognitive development, communication, and social interaction [1,2,3,4]. 

Childhood deafness is a challenging concern for otolaryngologists, audiologists, speech therapist, and neuropsychologists because it affects different domains such as hearing, speech, learning, and cognitive abilities. Hearing loss affects the development of spoken language, and all other language-mediated social processes, including the neurocognitive and emotional processes such as social understanding, which is a fundamental aspect of social learning [5,6].

Depending on the severity of the hearing loss, hearing aid (HA) or cochlear implant (CI) can be used to restore hearing. In general, HA is prescribed for hearing thresholds within 70 dB, while CI should be implanted above this threshold. The ASHA guidelines suggest the use of hearing aids starting from mild hearing loss (26 to 40 dB) up to moderately severe (56 to 70 dB), while for severe (71–90 dB) to profound (90+ dB) hearing loss cochlear implant should be used [7]. 

Any device for supporting reduced hearing functions, or restoring hearing in case of deafness, could be considered a valid tool to help developmental functions in children with hearing impairment and avoid deterioration of the cognitive function in adults with hearing loss [8,9].

To date, few studies have measured the intelligence in severe to profound deaf children with CIs. Traditionally, the outcomes for children with CIs have been focused on speech perception, oral language skills, and literacy by ignoring the important role that cognition plays in facilitating these functions. In a previous study, we showed the efficacy of hearing aids to improve memory and school performance in patients with unilateral hearing loss [8]. Some researchers compared implanted children with a healthy control group [10]; others evaluated the effect of speech rehabilitation alone compared to cochlear implantation without speech rehabilitation [11]. In general, these studies investigated the benefits of cochlear implantation on hearing, speech production, speech perception and language development, but none of these analyzed the effect of hearing rehabilitation on memory and cognitive/intellectual skills.

Intellectual quotient (IQ) is a number that represents a person’s ability of using their knowledge; it includes the capacities of acquisition, comprehension, storing, analyzing, synthesizing, reasoning, producing, and communicating.

A meta-analysis performed in 2016 showed that the IQ of subjects with unilateral hearing loss was lower the normal range [12]; the authors also identified verbal IQ lower than normal. Recently, Okely and colleagues performed a long longitudinal study analyzing IQ during the life course from childhood to middle age and they found that people with hearing Loss (HL) had lower IQ than healthy people [13]. The authors did not analyze the impact of hearing rehabilitation, nor compared patients with HL with hearing aids and those without. 

The IQ includes a series of skills like comprehension and analysis of speech, and full understanding of its content, so an auditory deficit, especially in childhood when the brain has its maximum development, might determine a hypo development of some brain areas with consequent reduction of the IQ. We speculate that this phenome might be observable in children only because childhood is the moment of maximal expansion of IQ capacities. In the literature it has been observed that hearing loss in elderly was correlated with brain atrophy and cognitive decline but not necessarily with the decrease of IQ scores [14].

Because the IQ includes also auditory skills like comprehension and analysis of the speech and its content -for example the distinction between vocal and consonants with similar sounds (“p” and “b”)- we want to evaluate if the use of CI or HA, which are two different technologies, can allow to obtain similar results in terms of IQ.

In theory, CI should allow the recovery of better hearing performance than HA, but this is today only speculative. 

The purpose of the present prospective analysis was to compare the effect on the cognitive function of CI vs. HA on a sample of children in developmental age affected by severe sensorineural hearing loss (SNHL) to understand if these two different devices can be equally efficient.

## 2. Materials and Methods

This prospective study included 21 children recruited at tertiary referral center from January 2021 to July 2023. The patients (10 girls and 11 boys) aged 6.5 to 8 years, with severe to profound deafness and bearers of a bilateral CI or bilateral HA were included in this study. This study was conducted in accordance with the Helsinki Declaration of Human Rights. Parents signed a written consent form to authorize the inclusion of their child in this study and the use of the clinical data for scientific purposes. This study was approved as an observational study by the Internal Review Board (IRB), number SB 13/21.

Because this study was conducted on children whose wellness and health must be protected, the IRB approved this project without the inclusion of a sick not-treated control group (children with hearing loss without any type of treatment). For ethical reason having an untreated group was not possible.

All children included in this study were evaluated by ABR and Pure Tone Auditory test (PTA) before the hearing rehabilitation (CI or HA) and with PTA after the use of hearing devices.

ABR testing was performed using Eclipse (Interacustics (https://www.interacoustics.com; accessed 1 October 2021) in the clinical automatic modality. The operator delivered a single click stimulus, starting at 100 dB and decreasing to 10 dB. The ABR threshold was set to the average hearing level of both ears at 2/4 kHz: 40 dB hearing level (HL) used a click stimulus at 70 dB normal hearing level (nHL), 40–60 dB HL used a click stimulus at 80 dB nHL, and 460 dB HL used click stimulus at 90 dB nHL. Contralateral masking was used if asymmetric responses were observed. ABR results could be absence of waves, presence of waves with altered amplitudes and/or latencies, normal ABR waves.

The PTA was performed at T0 earphones in a silent cabin to evaluate the level of hearing loss. The sound stimulation started from 10 dB, with increases of 10 dB and decreases of 5 dB, to confirm the sound perception. The impulse for each frequency tested (250, 500, 1000, 2000, 4000, 6000, and 8000 Hz) was sent to the studied ear three times, following the method described above. At T1, it was performed with the same modality but without wearing earphones (free field auditory test).

Cognitive ability and IQ were measured using the Wechsler Intelligence Scale for Children—Fourth Edition WISC-IV [15,16], a test specifically designed to assess the cognitive ability of children aged 6 years to 16 years and 11 months.

The neuropsychological assessment was carried out at the Department of Prosthetic Surgery for infantile deafness on a single day dedicated to in-depth diagnostics and was performed by a neuropsychologist with over 15 years of experience with children. 

The WISC-IV test assesses cognitive abilities with a particular emphasis on fluid intelligence, working memory and processing speed. The test is a good predictor of child’s academic performance, because it provides an overall assessment of general cognitive functioning, with the calculation of the child’s overall intelligence quotient (QIT) and the intellectual capacity. The test measures (i) visual processing, (ii) crystallized intelligence, (iii) fluid reasoning, (iv) short-term memory, and (v) processing speed.

The data collected using this scale were compared with the normal ranges included in the assessment to determine whether the IQ and subtests were normal (Table 1).

Inclusion and exclusion criteria were applied. 

We included all patients whose parents agreed to participate in this study, children who had parents with at least a high school degree and good socio-economic conditions.

We excluded patients with chronic diseases, neurological or neuropsychological disorders, under treatment with drugs, with poor socio-economic conditions, and whose parents disagreed to participate to this study.

All the children were treated at the hospital (outbound patients) with speech therapy three times a week, with sessions lasting 30 min each, and advance vocal training (AVT) once a week every two weeks for 12 consecutive months. The same speech therapist (VI) with over 15 years of experience followed the children. 

The following data were collected for each child: age in months, gender, ABR results at T0, PTA at T0, and T1 genitive test results test at T0 and T1, IQ at T0 and T1.

### Statistical Analyses

The double-tailed τ-test was used to analyze the difference in age between cochlear implant and hearing aid users before and after one year of hearing rehabilitation. The same test was used to evaluate the changes in the IQ before and after treatment in the two groups. One-way ANOVA was used to compare the subtasks of the WISC-IV test both within group and between groups. The Chi-square (χ) test was used to evaluate the differences in the improvement in specific subtasks between CI and HA in cases where the scores at T1 were below the normal range. P was considered significant < 0.05. All statistical tests were performed with Stata^®^.

## 3. Results

### 3.1. General 

A total of 21 children was recruited in this study. None of the children was affected by any disease, except hearing loss, and none of them used drugs before or during the period of the follow-up. All patients performed exactly the same speech rehabilitation therapy. In addition, 100% of the children had their auditory threshold at 20 dB average at least (normal hearing range) after use of the hearing device. The cognitive tests were performed without visual reinforcement exactly as generally performed in healthy subjects. 

### 3.2. Children with Cochlear Implant

In total, 11 children (5 girls and 6 boys, age average in months 88.3 ± 11.9) underwent surgical implantation with bilateral cochlear implants [Cochlear (five patients) or Medel (three children) or Advance Bionics (three subjects)] within 14 months from birth (average 11 ± 1.6; CI95%: 9–14). The children had profound bilateral hearing loss (deafness) with an average threshold of 110.3 ± 23.8 dB (CI95%: 90–120) (Table 2). All children improved their IQ 12 months after hearing rehabilitation. Five children had a low IQ at T0. Of these, one girl recovered to the normal range after 12 months of rehabilitation and 4 still presented IQs under normal at T1 (grey bold in Table 2). 

Despite the improvement at T1 in the IQ and subskills at 12 months, the scores were not statistically significant scores (ANOVA: *p* > 0.05). It should be noted that the verbal performance and processing speed index remained under normal in three patients (27.3%) even after hearing rehabilitation. 

### 3.3. Children with Hearing Aids 

In this study, 10 children (five girls and five boys, average age 88.5 ± 9.2 months, were treated by bilateral digital hearing aids (two patients with OTICON OPN Play 1 BTE PP, two with 2 BTE PP, and six with Phonak Sky M90-SP), fitted within 12 months of diagnosis (average 8.3 ± 3.8) and before 36 months of age (average 18 ± 9.6; CI95%: 11–30). The mean bilateral hearing thresholds were 77.1 ± 9.9 (CI 95%: 70–93) (Table 3). All children improved their IQ from T0 to T1, although this improvement was not statistically significant (ANOVA: *p* > 0.05). Four children had IQ scores lower than normal at the baseline, of whom, one recovered up to reach a borderline score, and three children still presented IQ scores under normal range after hearing rehabilitation (grey bold in Table 3). Looking specifically at the subtests at T1, two children presented reduced verbal comprehension (20%), while the processing speed index was under normal level after treatment in 7 children (70%).

No statistically significant differences in chronological age were observed between the two groups (τ-test: *p* = 0.14) at T0 and T1. The mean age of the cochlear implant group was 88.3 ± 11.9 at T0 and 100.3 ± 11.9 at T1, and the hearing aid group was 88.5 ± 9.2 at T0 and 100.5 ± 9.2 at T1.

**Figure 1:**
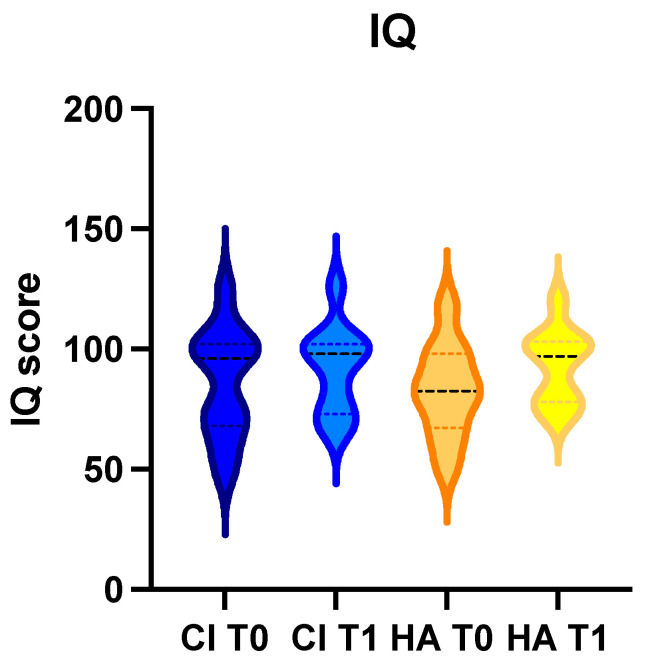
IQ averages (interrupted line into the figure) were similar in the two groups both before (T0) and after treatment (T1).

The comparison of the IQ did not show statistically significant differences between the two groups before and after treatment (τ-test: *p* = 0.4); the same lack of statistically significant value was observed when comparing the sub-tests. Children in the cochlear implant group had an average IQ of 86.4 ± 22.7 at T0 and 91.7 ± 18.1 at T1; patients in the hearing aid group had an average IQ of 83 ± 19.6 at T0 and 93 ± 15.6 at T1 (Figure 1).

The processing speed index was lower than normal at T1 with statistically significant value (χ: *p* = 0.03) in HA when compared to CI, meaning this specific subtest in HA was under normal range even after treatment.

## 4. Discussion

Overall, our study showed that the use of a hearing system, both CI and HA can benefit the IQ of children with severe hearing loss. The children were treated with one or the other auditory device based on the severity of their hearing loss and all subjects underwent early auditory rehabilitation. This study included patients aged between 6.5 and 8 years, who underwent hearing rehabilitation before language development. The IQs identified in the patients after treatment were consistent with previous research looking at the opposite side of the history, deaf people suffer from cognitive deficits in absence of adequate hearing rehabilitation [1,2,3,4]. 

Although at baseline the hearing thresholds of the CI users were worse than those of the patients with HA, respectively 110.3 dB (profound hearing loss) and 77.1 dB (severe hearing loss), the two groups did not show significant differences in their IQ scores after one year of hearing rehabilitation plus speech therapy.

The time of the hearing rehabilitation, as well as the correct rehabilitative device are fundamental to allow a normal development of intelligence. Moreover, it is extremely important to perform, especially in children with HA, regularly follow-up not to miss deterioration of the auditory thresholds that could make this prosthesis not perfectly performant for the patient’s needs. 

The analysis of IQ scores in the groups and their comparison with the normal range values matched for age, showed the persistence of IQs lower than normal both in CI and HA-treated children; this parameter remained under the normal threshold despite its improvement from baseline (T0). Subtests revealed some difficulties in the verbal comprehension and processing speed performance (PSI), which were under normal range despite recovery after hearing rehabilitation. In total, 70% of the children treated with HA had a PSI under normal, whereas only 27.3% of the children treated with CI had a similar result. 

Slowed PSIs have been observed in children with attention-deficit/hyperactivity disorders (ADHD) [17] and have been associated with altered working memory functions. Our previous studies showed that children with unilateral hearing loss, who underwent auditory rehabilitation, had slower working memory performance than normal because their cognitive performances were negatively affected by the excess of sounds [17]. We speculate that this phenomenon may also occur in deaf patients regardless of the type of hearing aid used; the sensitivity to the external sounds may be related to the brain characteristics of each child, making some children more sensitive than others to the stress of repeated sounds [17,18,19].

In addition, the delay in VU, PSI, and the IQ under normal range could be all related to other factors (environment, family support) that could significantly affect the children’s cognitive development [18]. Hearing rehabilitation alone could not be sufficient without modification of the additional influencing factors. Unfortunately, these additional factors were not investigated in this study, and this is an important limitation. 

The timing of hearing restoration, which was always before 36 months of age, is extremely important to maximize brain development; in fact, it is known that the earlier the treatment, the better the recovery, due to the dramatic increase in the synaptic connections between birth and 24 or 36 months of age [19]. 

The absence of differences in terms of cognitive development may indicate that CI works better than HA, as previously shown in the literature [4,6]; in fact, some of the patients in the HA group had moderate hearing loss (Table 3) at T0, which could have impacted the IQs at T1. Indeed, preservation of the auditory functions has been associated with better functioning of all brain connections [2,8,9], and has been shown that severe and moderate hearing loss affects brain development differently [11,20]. Children with mild-to-severe hearing loss tend to develop the right hemisphere of the brain more than healthy subject to compensate the auditory deficit, allowing them to present cognitive abilities like normal hearing subjects [21]. In any case, thanks to the child’s brain plasticity [22], the use hearing prostheses (CI, HA or bone anchored hearing aids) combined with speech rehabilitation [23] make possible to obtain the same QIs regardless of the severity of the hearing loss. 

Another important aspect to highlight is that although the use of HA is recommended for hearing loss within 70 dB [24,25], in this study we used this device even to treat severe hearing loss obtaining a real benefit for the patients. In fact, the results obtained in terms of IQs and sub-tests overlapping between the two groups. It is important to note, that in the CI group 4 children showed IQs score under normal level after 12 months versus 3 in the HA group. We speculate that this could be linked to better auditory threshold in the HA at T0. However, this finding was observed in very small sample of people and further studies with larger samples are needed before recommending HA prostheses as alternative to CI for hearing threshold above 70 dB.

If our results are confirmed on larger sample this finding could be important to reduce a series of risks related to the surgery that is necessary for CI. 

In fact, the use of HA instead of CI would avoid the risk specifically related to CI surgery [26,27] as well as the ones related to the surgery itself [28,29,30] and the anesthetic procedures [31]. Today both surgical and anesthesiologic procedures are safe, however, children always represent a fragile population [26,27,28,29,30,31] and their management needs careful attention. Non-surgical hearing rehabilitation solutions could also avoid the post-surgical stress and its negative impact on the rehabilitation [32], which is a fundamental aspect to recovery the hearing abilities [33]. 

We reiterate that the substitution of HA with CI for severe SNHL is only speculative and today the current international guidelines for cochlear implant use in children must be absolutely respected [34,35].

Our groups were homogenous for age without statistically significant differences between them. But there were several parameters that could have impacted the results. First, the auditory thresholds at the baseline; the partial preservation of some auditory functions might allow the normal development of the right frontal and precentral cortex [21]. On the contrary, the patients with worse auditory threshold might have an overactivation of the right precentral and parieto-occipital cortices to compensate sensory loss [21].

Then, despite the used devices are all extreme high-quality, some small differences between HA and CI could have impacted the results [36,37]. It is known that HA offers less benefit than implantable device [9] because it amplifies the sound without restoring the physiology of the auditory apparatus. Cochlear implant technologies allow selective stimulation of specific portions of the cochlea exactly as it happens in physiologic hearing [38]. As additional, some CIs seem better than other in terms of quality of sound [37]. These differences both in terms of devices used and technologies must be better investigated on larger samples. The comparison between specific mono brand devices, for example Sophono HA versus Medel CI, could be useful to understand the differences between CI. 

Finally, despite all children were implanted/treated before 14 months of age, some small differences between those implanted before 12 and after 12 months of age could be present [39], even if brain plasticity is quite similar under 15 months.

Our results, although preliminary, seem to confirm that the rehabilitation of the hearing function by the age of 8 years can support the development of normal IQs, independently of the rehabilitation technology used. Anyway, the device must be selected based on the patient’s hearing thresholds to maximize the cognitive recovery [9]. 

Despite the rehabilitation, some of the patients did not recover fully normal performance in very specific subtests (VU and PSI); this could be related to several additional factors rather than strictly to the hearing aids used. A strong psychological and sociological support should be considered for the families of deaf children to help them in the correct management of their children’s conditions. In fact, thanks to the high plasticity of child’s brain, the restoration of normal auditory function by hearing aids can allow children with hearing impairment to develop cognitive functions comparable to those children with normal hearing [8] especially if parents and family correctly support their child. As described above, the use of a hearing aid has a positive effect on hearing ability, especially in noisy conditions such as those that may occur at school. In this environment the correct stimuli can be absolutely helpful to increase brain plasticity and, consequently, IQs.

To summarize, we think that hearing rehabilitation directly improves hearing and directly and indirectly improves brain development.

### Study Limitations

This study has several limitations. Firstly, the small sample size, which only allows us to define preliminary results. Secondly, we did not include a control group; however, we compared the children’s IQ with already validated age-matched scores of normal subjects, and we believe that this made our results consistent. Finally, because several variables affect the development of IQ, despite strict inclusion and exclusion criteria, we could not be totally sure that all patients were exactly exposed to the same environmental stimuli. We are confident that the rigorous application of the inclusion and the exclusion criteria and the small sample size made the results robust. Additional studies using multivariate analyses of all findings that can impact the IQ must be conducted to fully understand the real impact of the auditory prothesization on these scores.

## 5. Conclusions

This study showed that hearing rehabilitation by CI or HA can be useful for IQs. Previous studies have shown the importance of hearing rehabilitation for language development and memory function. To the best of our knowledge this is the first study that analyzes IQs and subtests comparing children with severe hearing loss treated with two different hearing solutions. HA seems to be a valid tool even for hearing threshold > 70 dB, but this result must be considered preliminary due to the small sample size. It is important to note that all children included in this study were treated before the age of 14 months, so we recommend early treatment of hearing loss to support cognitive development. Due to the small sample size, although stratified by age, additional prospective case–control studies on larger samples and with longer follow-up are needed to confirm our data.

## Figures and Tables

**Table 1:** Reference parameters of Wisc IV assessment based on healthy children between 6 and 8 years of age.

Wisc IV	
Normal	85–115
Slight deficit	84–70
Moderate to severe deficit	69–49

**Table 2:** Characteristics of group treated by cochlear implants.

Gender	Age of Implant Months	ABR noWV	PTA (dB)	CA T0	VU T0	VPR T0	WMI T0	PSI T0	IQ T0	CA T1	VU T1	VPR T1	WMI T1	PSI T1	IQ T1
M	12	90 dB	90	112	72	89	85	79	75	124	90	85	94	91	80
M	13	90 dB	90	80	126	139	130	68	124	92	126	140	130	70	126
M	14	90 dB	140	81	100	98	102	100	104	93	100	100	103	100	101
F	11	90 dB	90	84	54	72	79	71	68	96	76	90	95	90	90
F	13	90 dB	140	95	62	87	73	56	59	107	70	95	75	60	65
F	14	90 dB	140	108	62	85	82	65	74	120	62	84	80	64	73
M	12	95 dB	98	81	102	100	103	103	102	93	102	100	103	103	102
M	13	90 dB	90	74	104	100	91	88	96	86	104	100	97	94	99
M	14	100 dB	100	83	60	71	55	59	49	95	72	87	76	74	70
F	12	90 dB	95	88	100	104	91	91	97	100	100	104	97	91	98
F	13	100 dB	140	85	106	106	97	91	102	97	106	106	100	100	105

dB: decibel; ABR: auditory brain response; PTA: pure tone auditory test; CA: chronologic age in months; noWV: absence of wave V; VU: verbal understanding; VPR: visuo-perceptual reasoning; MI: memory index; PSI: processing speed index; IQ: total intelligence quotient. In grey are the patients who presented abnormal IQ scores.

**Table 3:** Characteristics of group treated by hearing aids.

Gender	Age Hearing Aids Months	ABR eWV	PTA (dB)	CA T0	VU T0	VPR T0	WMI T0	PSI T0	IQ T0	CA T1	VU T1	VPR T1	WMI T1	PSI T1	IQ T1
M	14	90 dB	93	80	108	71	97	65	84	92	110	92	115	80	104
M	15	70 dB	70	84	110	122	124	67	118	96	120	110	100	70	120
F	13	90 dB	98	81	90	87	109	56	81	93	102	100	110	64	94
F	15	70 dB	73	108	75	90	85	72	78	120	77	92	87	74	82
M	16	70 dB	72	79	60	89	73	65	69	91	70	98	82	71	79
M	1	73 dB	73	89	62	74	55	59	51	101	78	95	79	68	75
M	13	72 dB	74	92	76	80	64	62	62	104	84	87	70	68	71
F	12	70 dB	71	82	98	100	85	79	89	94	102	108	94	91	100
F	11	74 dB	75	96	104	100	94	88	97	108	108	104	100	94	103
F	13	70 dB	72	94	108	106	94	88	101	106	108	106	97	91	102

dB: decibel; ABR: auditory brain response; PTA: pure tone auditory test; CA: chronologic age in months; eWV: evocable wave V; VU: verbal understanding; VPR: visuo-perceptual reasoning; MI: memory index; PSI: processing speed index; IQ: total intelligence quotient. In grey are the patients who presented abnormal IQ scores.

## Data Availability

Data are available under request to the corresponding author.
